# Comparative effects of alternate tobacco products and traditional tobacco smoking on various organ/systems: A guide to regulators and health policy makers

**DOI:** 10.1016/j.toxrep.2025.102100

**Published:** 2025-08-21

**Authors:** Saheed Olanrewaju Afolabi, Solomon Olagoke Olaoye, Amase Nyamgee, Anoka Ayembe Njan

**Affiliations:** a Department of Pharmacology and Therapeutics, Faculty of Basic Clinical Sciences, University of Ilorin, Nigeria; b Department of Medical Microbiology and Parasitology, Faculty of Basic Clinical Sciences, University of Ilorin, Nigeria

**Keywords:** E-cigarette, Nicotine, E-juice, Respiratory system, Cardiovascular system, Gastrointestinal system, Reproductive system

## Abstract

Traditional Tobacco smoking (TTS) is globally known as the single largest avoidable risk factor for a broad range of diseases. It is believed to be a “pandemic” because of its deleterious global impact on public health. Over a century ago, there has been a wide spread of tobacco cigarettes, originating particularly from the Americas; and most recently alternative tobacco products, such as heated tobacco products (HTPs), nicotine pouches, e-cigarettes, etc. This is due to the perceived safety of the latter. Data for this review were gotten through a rigorous search of scientific literatures on PubMed, Elsevier, Google scholar and Scopus. Unbiased findings on these search engines were included in this study, while claims that are unscientific were excluded. The deleterious health effects of e-cigarette aerosols (ECA) have been linked largely to the e-juice or e-liquid which majorly contains nicotine, flavorings, propylene glycol and other unregulated additives. In the respiratory system, TTS and ECA cause increase in pulmonary macrophage count and higher cell influx. However, TTS caused a higher lipid peroxidation judged by an increase in malondialdehyde (MDA) level. ECA caused a shift in the histo-architecture of the lungs, featuring an increase in volume density of the alveolar space which is associated with Chronic Obstructive Pulmonary Disease (COPD). Studies involving the cardiovascular system explored the e-liquid constituents such as nicotine, linked to atherosclerosis; cardiac tissue remodeling and cardiotoxic thermal metabolites of propylene glycol. On cardiac tissue remodeling, ECA caused significant increase in angiogenesis in mouse heart tissues, coupled with increase collagen production but not tissue fibrosis. This suggests that acute exposure to ECA did not adversely affect contractile functions or fibrosis. However, this was contrary with TTS, which showed inhibition of angiogenesis and induction of cardiac fibrosis. The increasing use of ECA amongst young adults showed more tendency for neurological defects when compared with TTS (since its consumption is reduced), this is mainly due to combinatory neurotoxic effects of nicotine, flavorings, formaldehyde, etc., causing a negative effect on cognition and attention span.

Putting these together, further research needs to be carried out on long-term safety of e-cigarettes, while national health regulators and policy makers should provide informed policies on the use of e-cigarettes and other alternative tobacco products.

## Graphical Abstract

**Figure:**
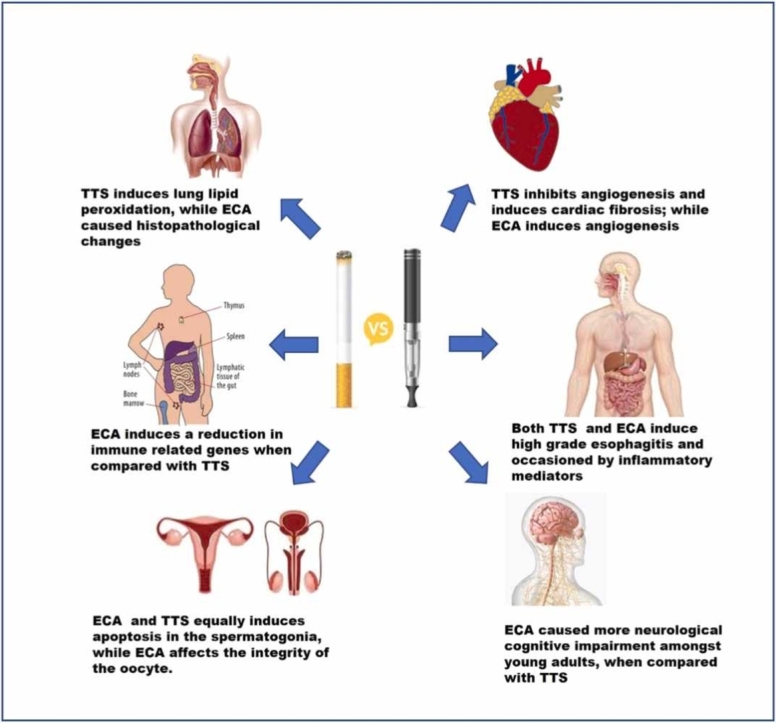

## 1 Introduction

### 1.1 Tobacco smoking

**Fig. 1:**
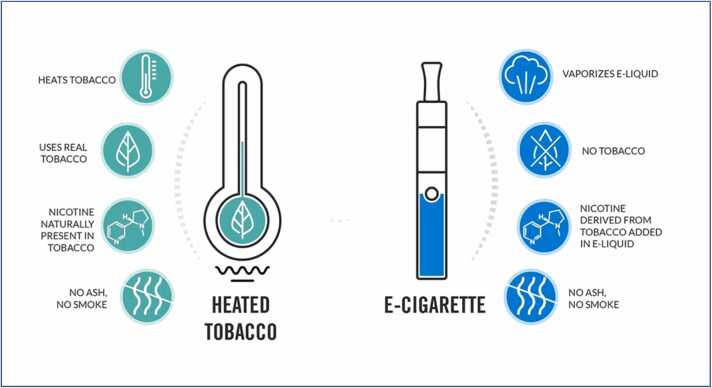
Content and mode of using traditional tobacco and E-cigarette**; (**Adapted from Philip Morris International: https://x.com/InsidePMI/status/1194653651765215232).

Briefly, tobacco smoking is the act of consuming (via inhalation) the byproduct of combusted tobacco. The process may involve inhaling as it is done with cigarette or released from the mouth as it is seen with the use of pipes and cigars [117]. Tobacco smoking dates back to 5000 – 3000 BC originating in South America and Mesoamerica [24]. A major psychoactive component of tobacco is nicotine, which acts on the CNS to create a relaxing and pleasurable euphoric feeling, that comes along with dependence and subsequently addiction [58] (Fig. 1.).

Tobacco leaves are dried, pulverized and processed in various ways, with resulting products that can be smoked, applied to gums, in the case of nicotine gums or inhaled as “snuff” [76]. In the process of commercializing the tobacco product, several additives are included, such additive includes sweet hay, rose oil, fruity aromatic flavors, etc., with the intent to increase acceptability, addictive potency and improve palatability [73]. Many constituents of cigarette smoke, chiefly nicotine, increases heart rate, alertness and reaction time, as adjudged by its consumers. The most popular means of consuming tobacco is via smoking, many smokers begin during adolescence and continue almost throughout their lifetime [21], [25].

A study on first smoking experience of 7th Graders discovered that the most common factor enhancing tobacco smoking is tobacco product advertisement [102], [39]. Other compounding factors include peer pressure, peer acceptability, parents smoking, amongst others. At the onset of tobacco smoking, the act is stimulated by a perceived pleasure acting as “positive energy” [89], whereas the desire to respond to social peer pressure usually offsets the unpleasant initial phase of nausea and coughing. Several reports suggests that most cigarette consumers started smoking at adolescence [25].

### 1.2 Alternate tobacco products (Atps)

Alternate tobacco products include all tobacco-containing and nicotine-delivery systems that are manufactured, marketed and consumed as alternatives to conventional cigarettes [91]. They are also referred to as novel tobacco products or non-conventional tobacco products and are now of great relevance in the context of global tobacco harm reduction debates [56], consumer preferences, regulatory protocols and tobacco products marketing [34].

Various forms of these products exist with varying constituents and ingestion modes. These include:

#### 1.2.1 Smokeless tobacco products

They are tobacco products that are consumed without any form of combustion or heat [72]. They are either chewed, sniffed or sucked and they include:

##### 1.2.1.1 Snus

Snus is mostly found in the Scandinavia countries particularly Sweden where it is produced by Swedish Match, a tobacco company under the Philip Morris International [42]. It is manufactured as a pasteurized moist powdered tobacco often placed in pouches of small sachets and consumed by placing it between the upper lip and the gum [42]. Its identified constituents include nicotine, nitrosamines, polycyclic aromatic hydrocarbons and heavy metals.

##### 1.2.1.2 Chewing tobacco

Chewing Tobacco (CT) are raw tobacco leaves which are packed with or without additives and are chewed by users who are mostly residents of South Asia [48] and the United States of America. They are produced by local manufacturers and industries such as *Manikchand* and *Dharampal Satyapal* Group in India. The constituents of CT include nicotine, slaked lime (often added as additives in South Asia), catechu, areca nut and sweeteners.

##### 1.2.1.3 Snuff

Snuff are powdered tobacco leaves that are consumed via the oral or nasal routes [29]. They come either as moist or dry products and are placed on the tongue, in between the cheek and the gums or sniffed via the nostril by users. Users of snuffs are found in Africa, United states of America and the United Kingdom. They are prepared in small quantities by local producers and in large quantities by tobacco industries such as the United States Smokeless Tobacco Company (Altria) and the British American Tobacco. The major components of these smokeless tobacco products (STPs) include nicotine, tobacco-specific nitrosamines (TSNAs), sugars, humectants, sodium ions, chloride ions and ash [82].

##### 1.2.1.4 Dissolvable tobacco

This is produced either as gum, sticks, lozenges or strips and are specially designed to dissolve in the oral cavity where they are placed by users [9].

#### 1.2.2 Nicotine pouches

These are novel nicotine-containing products. They do not contain tobacco but slowly releases nicotine when placed in the mouth of its users.

##### 1.2.2.1 Heated tobacco products (HTPs)

HTPs are electronic devices designed to heat tobacco at low temperatures lower than that needed for combustion but in the process release nicotine containing aerosols for inhalation by the users. These include:

##### 1.2.2.2 IQOS

This product is manufactured by Philips Morris international [32] and consists of a holder or charger which is connected to a disposable stick referred to as HEETS or Heat Stick. The HEETS contains processed tobacco and glycerin. The holder heats the content of the HEETS to release nicotine containing aerosols for inhalation by users. The chemical constituents IQOS include nicotine, glycerol, volatile organic compounds, carbonyls and nitrosamines. These products are promoted as a reduced-risk products and are largely marketed and used in Europe, Asia and the Americas.

##### 1.2.2.3 Glo

This is British American Tobacco (BAT) company’s variant of the IQOS. Asides Glo, other grades of the product are also manufactured by BAT and these include glo Pro, Hyper, Hyper+ and Hyper X2. These devices consist of a handheld chamber which houses a lithium battery designed to power a second segment called the heating chamber. The heating chamber is connected to prefilled tobacco tubes known as Neostiks for heating at temperatures that are far lower than required for combustion.

#### 1.2.3 Electronic nicotine delivery systems (ENDS)

Unlike the HTPs, the devices under this category do not contain tobacco but are capable of delivering nicotine for inhalation as vapors and aerosols. They are also known as Electronic Cigarette (E-Cigarette) or vapes and are battery powered devices that vaporizes a nicotine-containing solution called e-liquid or e-juice. They consist of a cartridge or tank within which the e-liquid is loaded, an atomizer which serves as the heating element and a power source containing a battery that powers the atomizer. Upon heating by the atomizer, the liquid cartridge releases vapors which cool to give a combination of aerosols of tiny droplets, vapors and air (Fig. 1.). The e-cigarettes contain the e-liquid which majorly contains nicotine, propylene glycol, glycerin, flavorings and aldehydes [50]. The available e-cigarettes include Cigarlike which resemble conventional cigarettes e.g. Blu, Vape pens with refillable tanks, Pod systems e.g. JUUL and Mods. They are manufactured by tobacco giants such as JUUL Labs (USA) which is owned in part by Altria, RELX (China) which dominates the Asian e-cigarette market and NJOY which was recently acquired by Altria. These products come with different flavors and constituents.

#### 1.2.4 Nicotine pouches and lozenges

These are tobacco free pouches and tablets containing plant-derived or synthetic nicotine [97]. Examples include ZYN, VELO and On. These products are manufactured by companies such as Swedish Match, BAT and Altria and are available in the USA, Scandinavia and Europe.

#### 1.2.5 Waterpipes

Waterpipes include but not limited to Shisha and Hookah (which contains nicotine, lead, cadmium, etc.) is gaining major subscription across the world especially in Africa, Middle East, and South Asia. These are charcoal-heated flavored tobacco inhaled through a water bowl [45]. The product of these combustion includes carbon monoxide, tar, and some aldehyde derivatives [95]. They are manufactured by local producers and industries such as AI Fakher (UAE) and Nakhla (Egypt).

## 2 Epidemiological trends and patterns of Atps consumption

The World Health Organization (WHO), Global Adult Tobacco Survey (GATS), Global Youth Tobacco Survey (GYTS) and various regional surveillance systems have documented several epidemiological data which provide substantial insight into the use of ATPs. The most commonly used ATPs are the E-cigarettes with user prevalence as high as 20 % among adolescents in United States of America, United Kingdom and some countries in Europe [1].

The WHO estimated a user population of 82 million of e-cigarette in 2021 against the 7 million user population in 2011 [67]. In 2023, about 2.13 million (7.7 %) middle and high school students in the United States of America reported current use of Vapes [20]. Though the use of Vapes is higher in high income countries (HICs) the expanding market of the product is notably encouraging higher prevalence of consumers in the low and middle-income countries (LMICs).

Over 20 % of adults in the South Asia countries of India and Bangladesh as well as a sizeable pool of Africans are also users of smokeless tobacco due to socio-cultural acceptance especially in the rural communities and particularly among women. The national prevalence of the use of these products ranges between 20 % and 30 % in India and Bangladesh [66], [70]. Of the approximately 83 % of the global chewing tobacco users who are reportedly residents of South Asia, the user population in India and Bangladesh is put at 185.8 million and 25.7 million respectively [48]. The use of HTPs is particularly high in Bulgaria, Japan and South Korea as over 10 % of the adult population are estimated to consume these products regularly [107]. In most of the aforementioned regions, the use of ATPs has significantly reduced the subscription to traditional smoking. However, the incidence of dual-consumers (consumers of both ATPs and traditional cigarette) is on the rise [33], [94].

The European and Northern America market of HTPs is also said to be increasing due to the various marketing strategies of the producers. The use of Waterpipes is particularly of public health concern in Africa, Middle East and South Asia [4]. Data from GTYS particularly identified Jordan, Egypt and Lebanon as countries greatly contributing to the consumer prevalence of these products. The lifetime use rates of these products ranges between 10 % and 40 % in urban populations in North America and the Middle East [22]. The consumption of Nicotine pouches and other novel oral nicotine products are also on the rise in Europe and North America particularly among male youths.

## 3 Regulatory policies governing the use of Atps

The increasing use of ATPs is of great public health concern as it poses significant regulatory challenges particularly as these products are marketed as harm reduction tools despite their potential as gateway to nicotine dependence particularly among the growing youth population across the world [18].

Regulatory policies concerning ATPs vary from one country to the other and this reflects the socio-cultural acceptance, tobacco control maturity, public health priorities and tobacco market influence on the respective national economy. The available regulatory policies aim to protect public health, prevent youth initiation, prevent unnecessary exposures to non-users, ensure product safety, support smoking cessation, and prevent dual usage of cigarette and ATPs [31]. The WHO framework Convention on Tobacco control is the cornerstone of global tobacco regulation [49]. Though the initial treaty of 2003 did not explicitly address the burden of ATPs, subsequent Conference of Parties (COP) took far reaching decisions about ATPs including the application of similar regulations used for conventional cigarettes, banning the advertisement, promotion and sponsorship of ATPs, prohibiting misleading harm reduction claims, and implementing taxation policies to limit accessibility and affordability of ATPs [49].

The European Union Products directive (TPD) which regulates the use of e-cigarettes and other novel tobacco products limits the concentration of nicotine in any of these products to 20 mg/mL, and warns that such products must be manufactured with necessary health warnings. It also placed restrictions on some advertisement and mandates the disclosure of the constituent of these products [111].

In the USA, regulatory terms similar to those on tobacco applies to ENDS and HTPs use under the Family Smoking Prevention and Tobacco Control Act of the FDA. Manufacturers are therefore mandated to undergo premarket Tobacco Products applications to prove the appropriateness of their products for the health of the public. In 2022, the US. FDA proposed rules prohibiting menthol cigarettes and flavored cartridge-based cigars to reduce their appeal to the youth [47], [108]. Snus is however approved as a modified risk Tobacco Product in the USA. Though allowed with restricted advertisement, the sale of ENDS in Canada must be with strict health warnings by the manufacturers [10]. There is also a strong regulatory stance on ATPs in the Asia-Pacific. India enacted the *Prohibition of Electronic Cigarettes Ordinance* which is a comprehensive e-cigarette ban bill in September 2019 and this bill was subsequently signed into law by December, 2019 to prohibit the manufacture, storage, transportation or distribution, advertisement, sales or purchase of e-cigarettes in the country [5].

Singapore also enacted the Tobacco (Control of Advertisement and Sale) Act to enforce a strict ban on the importation, sales and possession of e-cigarette while Thailand did the same in 2014 [69]. The violation of e-cigarette prohibition law in Thailand attracts a 10-year imprisonment or a fine of 30,000 baht or both. However, the regulation of smokeless Tobacco Products is extremely variable. Japan permits the use of HTPs and hence its occupation of the larger part of the tobacco market but placed a strong ban on the use of nicotine-containing e-cigarettes. There is a restrictive regulation of ATPs in Australia aimed at discouraging youth uptake as well as encouraging smoking cessation among the populace. It however allows a prescription-based use of vaping products. Though the regulation of ATPs is gradually evolving in Africa, the ATP market was left unregulated a for a very long period of time. These countries are gradually framing Tobacco Control Acts that align with the WHO FCTC but are faced with serious implementation challenges due to inadequate enforcement mechanisms thereby weakening the regulatory policies in these countries [44].

South Africa and Kenya are developing some frameworks aimed at regulating the use of ENDS and HTPs. Sudan has in principle prohibited the use of smokeless tobacco but its enforcement is still poor. There is also a big challenge in the uniformity of surveillance and systems for monitoring and evaluation of the market situation.

The major challenges in the regulation of ATPS across the countries of the world stems from: the lack of an all-encompassing data on the health impacts of these products hence the creation of an evidence gap, market and marketing strategies influence on regulatory policies, dual use of ATPs and conventional cigarettes. Although the use of ATPs is not currently increasing amongst young adults in many regions, it still poses a major public health concern. This is primarily due to the expanding internet marketing of the products leading to the bridge of national bans and restrictions.

## 4 Effect of traditional smoking and E-cigarettes on biological systems

### 4.1 Respiratory and pulmonary system

Traditional tobacco smoking (TTS) exposes its consumers to over 7000 chemicals [110], [68], which are mainly products of thermogenic degradation and partial combustion of tobacco cigarettes [104]. Public health findings and toxicological data have reported that inhalation exposure to tobacco smoke is pulmonotoxic, leading to chronic bronchitis, asthma [27], [7], and the onset of chronic obstructive pulmonary disease (COPD) [36].

TTS is the main cause of lung cancer, and highly contributory to initiation of several other malignant tumors [57]. This has been linked to the presence of several chemical initiators of carcinogenesis, mainly polycyclic aromatic hydrocarbons (PAH, about 510), nitrosamines, and several other volatile organic compounds [77]. These established health hazards have led to the production of alternative tobacco and nicotine products, that may assist people in quitting smoking. These include heated tobacco products (HTPs); electronic nicotine delivery system (ENDS), such as e-cigarettes or popularly called vaping; Nicotine patch, etc. Although, Public Health England in 2015 stated that e-cigarette use (vaping) is 95 % safer than regular smoking and the use of vaping products is about 95 % less likely to result in death from tobacco use [114], [83], there is need to harvest comparative studies that deals with more specific, chronic, preclinical and clinical data on both forms of tobacco use.

In addition to this, there are hypotheses that these alternative exposure to tobacco products mitigates some pulmonary toxicities observed with traditional smoking [112]. These perceptions have led to several researches exploring the safety or otherwise of these alternative consumption methods. For instance, [109], observed in a study on COPD (which is characterized by a chronic inflammatory process in the lungs, coupled with the renin-angiotensin system) that cigarette smoke extracts from traditional cigarettes (CSE) caused higher cytotoxicity and higher oxidative stress levels than extracts from heated tobacco product extract (HTPE) in two lung cell lines (Calu-3 and Beas-2B). However, both CSE and HTPE triggered oncogenic stimuli such as RAS activation, NF-kB inflammatory pathway activation, MAPK activation, thus resulting in carcinogenesis.

**Fig. 2:**
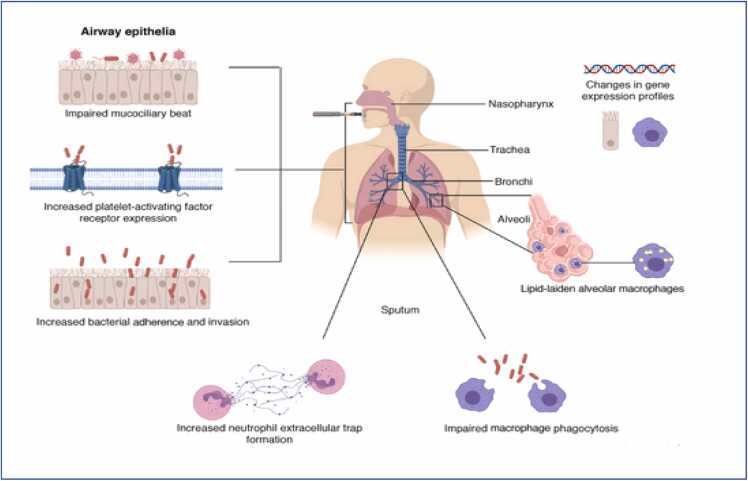
Multiple mechanisms delineating the toxicity of E-cigarette in the respiratory system (Figure adapted from [86]).

Another comparative study involving a short-term exposure to e-cigarette aerosol (ECA) and cigarette smoke in male mice, showed that both exposures resulted in increase in macrophage count preceded by a higher cell influx into the airways. Exposure to cigarette smoke however displayed a higher pulmonary lipid peroxidation product, malondialdehyde (MDA); both exposures promoted increased levels of interleukin 17 (IL-17). However, e-cigarette vapor led to a shift in lung histoarchitecture, featuring a higher volume density in the alveolar space (lipid-laden alveolar macrophages; Fig. 2), when compared to cigarette smoke. Therefore, this study inferred that short term exposure to e-cigarette vapor promotes acute inflammation that compares with cigarette smoke when assessing ventilatory parameters animal models [36].

A human study carried out to assess the acute respiratory response to e-cigarette use in exclusive e-cigarette users and dual users (traditional and e-cigarette consumers showed that short term exposure to e-cigarette caused a decrease in the concentration of nitric oxide in exhaled air (FeNO) and increase in airway temperature, while there was only a decrease in airflow in tobacco smokers and dual users, suggesting that the pattern of respiratory response was not particularly different in both groups [23]. [19] reports on the erroneous “less harmful” status ascribed to the use of e-cigarettes. This they portrayed by delineating the possible mechanisms underlying e-cigarette or vaping associated lung injury (EVALI).

EVALI is a disorder characterized by respiratory failure with an intense inflammatory response [71]. In 2020, the United States recorded over 2800 morbid cases and over 6o deaths as a result of EVALI outbreak. This lung injury was linked to vitamin E acetate (VEA) component of e-cigarettes which elicited tetrahydrocannabinol (THC) byproducts [74]. It was also proposed that EVALI should be looked out for in patients who have a history of vaping or consumption of other e-cigarette products because of its diverse presentation. Data obtained from rigorous internet search and over 40,000 social media posts between 2008 and 2015 suggests that many symptoms of EVALI have been reported online for at least 7 years in e-cigarette product users [65]. Therefore, these studies suggests that e-cigarettes have a worse acute toxicity than regular tobacco smoking, although there is still paucity of data on their long-term toxicity.

### 4.2 Cardiovascular system

Cardiovascular disorders (CVD) and heart related pathologies remain the major cause of global mortality and morbidity, ranking higher than cancer and other infectious diseases [41]. In 2021, 20.5 million CVD-related deaths were reported, 80 % of these global statistics occurred in low- and middle- income countries such as Nigeria [105]. There is already an established cardiotoxicity profile of traditional tobacco smoking, presenting cases such as hypertension, congestive heart failure, cardiomyopathies, arrythmias, angina pectoris; and exacerbating already existing cardiac pathologies [120], [13].

Since its launch in the late 2000’s, there has been an increase in the sales of e-cigarettes in the USA and Europe. This is largely due to its “less-toxic” promotion and flavor variants. However, cardiovascular safety is a major concern with e-cigarette usage. E-cigarette emissions contains nicotine, nitrosamine, propylene glycol, flavorings, etc., which might have deleterious effects on cardiac health. The incidence and mortality of various types of CVDs have a modest growth among e-cigarette consumers coupled with the fact that the complexity of the e-cigarette content adds a layer of cardiac concerns. For example, nicotine has been reported to cause atherosclerosis, arrythmia, cardiac tissue remodeling [15], [43]; propylene glycol can produce cardiotoxic thermal metabolites such as formaldehyde, acetol, propylene oxide [103]. [100] reports the adverse effects of nicotine on myocardial functions after an infarction following exposure to ECA (containing 5 mg/mL nicotine). This loss of contractile functions was however not observed in the control animals exposed to ECA not containing nicotine. This lays further credence to the role nicotine plays, as a major component of E-cigarettes.

**Fig. 3:**
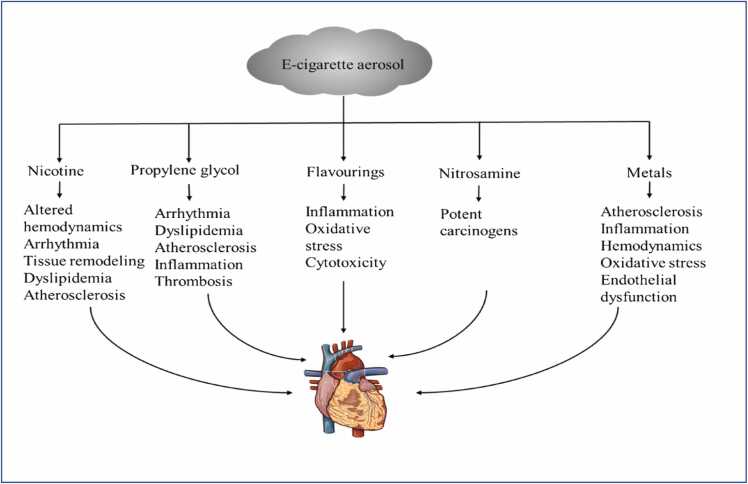
Cardiotoxic mechanisms of E-cigarette (Adapted from [123]).

Formaldehyde is a major cardiac toxicant causing atrio-ventricular block, arrhythmia, ventricular fibrillation, atrial fibrillation and ventricular tachycardia. Several of these toxicities have been adduced to oxidative stress, inflammatory mediation, endothelial dysfunction, platelet dysfunctions, dyslipidemia, hemodynamic effects, etc., (Fig. 3).

[38] carried out a comparative study on the chronic exposure of mice to e-cigarette aerosols (ECA) and tobacco smoke (TS). The goal was to assess biological markers linked to oxidative stress, inflammation and fibrosis. Also, cardiac proteomic analysis was used to identify shared and unique protein expression changes in e-cigarette aerosols and tobacco smoke. Cardiac parameters including heart rate, blood pressure, cardiac oxygenation, were significantly increased in TS exposure compared with the control; while in ECA exposure these parameters were not significantly affected, when compared to the control.

Myocardial damage due to exposure to ECA and TS was evaluated using the Transmission Electron Microscopy (TEM). This showed a discernable greater histopathology of the myofibrils, mitochondria and cardiomyocytes in the TS group, when compared to the ECA group. This manifested in mitochondrial membrane disruption, mitochondria swelling and partial degeneration of the myofibrils [14], [38]. Still considering myocardial health and tissue remodeling, a 2-week exposure to ECA showed alterations in cardiac functions via echocardiography [101]. The study showed that ECA exposure 3 h a day for 2 weeks (casual-model mimic), caused no marked effect on ejection fraction, but caused significant increase in angiogenesis in mouse heart tissues, coupled with increase collagen production but not tissue fibrosis. This suggests that acute exposure to ECA did not adversely affect contractile functions or fibrosis but induce cardiac angiogenesis, which could be beneficial in instances of myocardial infarction (MI). However, this trend is contrary with TS exposure, which features inhibition of angiogenesis. Also, the risk/benefits of increased angiogenesis as a result of ECA exposure needs to be further evaluated, since one of the hallmarks of cancer is upregulated angiogenesis [78].

### 4.3 Gastrointestinal system

The adverse effects of traditional tobacco smoking (TTS) on the gastrointestinal (GI) system are well established. It has been shown to predispose its consumers to altered bowel habits, such as irritable bowel disorder (IBD), exacerbated gastric reflux disorder, peptic ulcer disease (PUD), oral mucositis, gastric cancers, tongue discoloration, gingivitis, gum bleeding, nausea, vomiting, etc. Berkowitz et al. [115], [16]. Although tobacco smoking is believed to increase oesophageal acid exposure by increasing the number of reflux events [93], the effects of vaping on the gastrointestinal tract are not fully established.

This review seeks to put together the limited information available. A case study conducted by [93] examined a severe case of esophagitis associated with vaping, the result showed a daily exposure to ECA caused a high grade level of esophagitis. Histopathological assessment of esophageal biopsies showed tissue granulation with acute and chronic inflammation.

A systematic clinical survey on the acute effect of ECA on otolaryngology showed that an acute exposure to ECA leads to cough, intra-oral lesion, throat and mouth irritation [12]. A study on sub-acute effect of ECA inhalation on albino Wistar rat was explored to access the safety of ECA on the colon; and a follow-up study to check the after-cessation exposure effect. In the exposed group, the level of superoxide dismutase (SOD) was significantly reduced; while malondialdehyde (MDA), total nitric oxide (NO) and tumor necrosis factor-alpha (TNF-α) expression increased significantly in colonic tissue. Thus, suggesting oxidative stress as a possible mechanism underlying its colon toxicity. Histopathological findings on the colonic mucosa showed distortion and loss of its epithelial lining with an aggressive inflammatory cell infiltration [87].

Gastroesophageal reflux disease (GERD) is one of the most common gastrointestinal tract disorders [92]. Despite the strong association between smoking and GERD, the underlying mechanism(s) is still far-fetched [119]. A study on the prevalence of GERD on ECA exposure was assessed in university students in Jeddah, Saudi Arabia. Although e-cigarette consumption was more prevalent than tobacco smoking, there was no significant correlation between the consumption of e-cigarette and GERD [2].

A pilot study was carried out on e-cigarette use in adult patients with confirmed case of irritable bowel disorder (IBD). IBD is a GI disorder characterized by a chronic inflammation of the GI tract, caused majorly by both environmental and genetic triggers [6]. The links between TTS and IBD is well established as a major risk factor for Crohn’s disease and ulcerative colitis in current and former tobacco smokers. Whilst TTS greatly aggravates the onset and severity of Crohn’s disease, it seems to protect against the onset of ulcerative colitis and also reduce its severity [84], [90]. E-cigarette’s aerosol (ECA) inhalation increases expression of pro-inflammatory cytokines, induce gastrointestinal mucosal inflammation and impair the gut mucosal barrier [51].

### 4.4 Immune system

Tobacco smoking (TS) is a known trigger for impaired innate and acquired host defence responses and immune suppressive effects, upregulating inflammatory responses and deactivating immune memory cells [106], [28], [3]. The innate and adaptive immune systems affected by TS include memory T/B lymphocytes, macrophages, Natural killer cells (NK) and T helper cells [99]. Despite these established immunologic effects of TS, there is still limited information regarding the effect of ECA inhalation on the immune system.

A clinical gene expression study investigating the effect of ECA on nasal epithelia by collecting superficial nasal scrape biopsies, nasal lavage, urine and serum from E-cigarette users, cigarette smokers and nonsmokers showed a significant suppression of a large number of immune related genes in ECA consumers when compared with cigarette smokers and nonsmokers [81]. [122] reports summarily, the wide spread induction of inflammation, which compromises immune cell function, disruption of epithelial barriers causing susceptibility to viral and bacterial infections.

Another mechanism is the impairment of antiviral responses against pathogens like influenza A virus and SARS-CoV-2 [11], [53]. The widespread acceptance of ECA use as a cessation plan, with increasing exposure amongst young adults poses a growing global health concern. This is due to the fact that chronic use increases the risk of respiratory infections, consequent upon a suppressed immune mechanism and an increased potential for auto-immune disorders [122].

In an *in-vitro* setting, neutrophils were exposed to ECA extract, the expression of matrix metalloproteinase 9 (MMP-9) and interleukin 8 (IL-8) were measured using ELISA; while the expression of CD11b and CD66b were measured using flow cytometry. ECA extract increased the expression of all the markers, including an activation of mitogen activated protein kinase, MAPK, which is major oncogene expressed in several solid tumors [59]

### 4.5 Reproductive system

Traditional tobacco smoking (TTS) has been reported to be reprotoxic via mechanisms involving its adverse effects on sperm parameters, such as motility, morphology and count [8], [96]. This is believed to be due to the production of toxic metabolites that infringe on the integrity of the gonads [55]. DNA damage, disruptions in hormonal signals and oxidative stress, are key mechanisms behind these effects [96]. There are also reports of TSS having negative impact on female fertility via its teratogenic effect on the fetus and also the distorting hormonal homeostasis [40], [85].

Human studies on the effect of ECA on reproductive health are not very much. However, [88] in their review on the overview of e-cigarette on reproductive health states that despite lower levels of toxins in ECA when compared to TTS, the various components of the e-cigarette such as nicotine, flavorings, heavy metals still pose a threat to reproductive health.

In animal studies involving albino Wistar male rats, exposure to ECA caused increased apoptosis in spermatogonia and spermatocyte; alteration in morphology and function of seminiferous epithelium; disruption of steroidogenesis and general disorganisation of the testes [113], [116]. Unlike the trend observed on spermatozoa obtained from male Wistar rats there were no scientific data associating the effect of ECA use on oocyte genome integrity and intrinsic oocyte quality [30]. However, some studies infer that impaired ovarian function; decreased percentage of normal follicles; reduction in estrogen production; delayed embryo implantation, and high progesterone levels, resulting in a decreased offspring number was observed in rats exposed to ECA [118], [52]. In spite of these findings, more clinical studies are required to validate these findings.

### 4.6 Neurological system

Traditional tobacco smoking is considered one of the most preventable global cause of neurodevelopmental diseases [54]. Several neurotoxicity such as, neurodegeneration, addiction, mood disorders, depression, anxiety, etc., have been linked to chronic exposure to tradition cigarette smoke [17], [54].

**Fig. 4:**
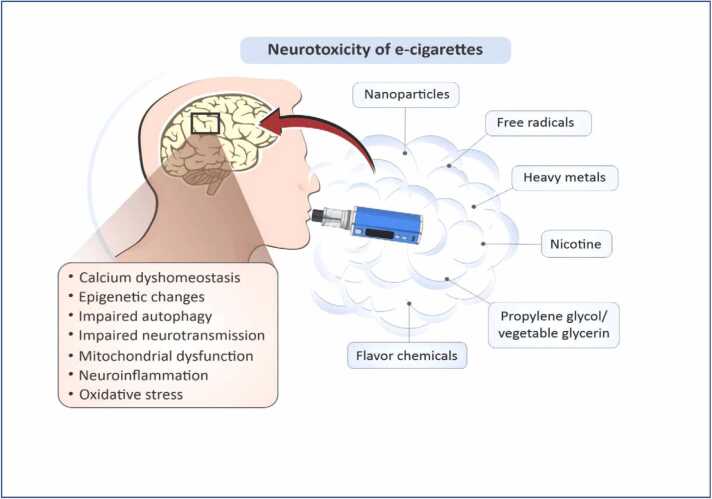
Neurotoxicity of E-cigarette Aerosol (ECA); (Adapted from [98]).

Nicotine-induced neurotoxicity cuts across various species ranging from animal models to clinical experiments, all adducing to the fact that adolescence ae more predisposed or vulnerable to nicotine neurotoxicity [75], [79]. Since nicotine forms the major component of the e-cigarette liquid (also called “juice”) [80], there is a major concern about exposure to ECA and its use as a substitute cessation plan for tobacco consumption (Fig. 4).

ECA-nicotine delivery system has been suggested to have a detrimental effect on juveniles, this is worrisome because of its increasing popularity amongst this demography since the CNS is largely still in a developmental phase, which is linked to experience-linked plasticity in the prefrontal cortex which is responsible for emotions, decision making and cognition [121], [37]. Nicotine exposure has been shown to increase neuronal activity, since nicotinic acetylcholine receptors (nAChRs) are critical control point for brain development, with higher expression during CNS formation and maturation [26], [35]; this suggests a greater effect in the brain of the adolescent in comparism to the adult. In addition to this, chronic exposure to nicotine during adolescence has grave consequences on cognitive pattern, causing reduced cognitive which could deteriorate into reduced attention span and enhanced impulsivity in adulthood (et al., 2020; Ruszkiewicz; [46]). Generally, ECA has been reported to cause neuroinflammation, oxidative stress, mitochondrial dysfunction, etc., all which are attributed to the constituents of the e-juice (Fig. 4).

## 5 International regulatory inputs

### 5.1 European commission scientific committee on health, environmental and emerging risks (EU-SCHEER)

In 2021, The EU-SCHEER set out to assess the risk associated with the use of e-cigarettes and they came forth with the following opinions:

1. On the respiratory system, particularly the respiratory tract, there is moderate risk of local irritative damage consequent upon the use of e-cigarettes. This is largely attributed to the cumulative exposure to nicotine, aldehydes and polyol. Although the reported incidence is quite low.
2. The long-term cumulative risk for cardiotoxicity is moderate; and the risk of carcinogenicity as a result of long term/ cumulative exposure to nitrosamines, acetaldehyde and formaldehyde is weak to moderate. Considering also the metal components of the e-cigarette aerosol, the risk of carcinogenicity was also found to be weak.
3. The general burden of evidence of other long-term adverse health effects, such as pulmonotoxic, neurotoxic and reprotoxic effects based on the hazard detection and human evidence, is weak. However, more consistent data are needed.
4. On flavouring, the committee reports that there are no specific data on any particular flavour in e-cigarettes causing long term deleterious health effects.
5. [61].

### 5.2 The Asia Pacific academic consortium for public health (APACPH)

The Asia Pacific Academic Consortium for Public Health (APAMT) has gathered information cutting across South-East Asia generally. The consortium reports that novel findings on the health effects of e-cigarette use have shown a strong link with increased risks of respiratory diseases, cardiovascular pathologies, irritation of bronchi airway and coughing. Region-specific surveys in Hong Kong and Indonesia reported a higher prevalence of upper respiratory tract symptoms in e-cigarette users.

Asides case reports from academic publications, diseases suspected to be e-cigarette-related, such as nose cancer, pulmonary edema, lung damage and dyspnea, were reported in Thailand, the Philippines and Malaysia in 2019. Between 2015 and 2018, there were reported incidence of burns, injuries and explosion to the faces of e-cigarette users in Brunei, Indonesia and the Philippines.

Some experts allude to the position that e-cigarette consumption is safer than combustible tobacco use. Although, there is still paucity of information on the chronic health impacts/effects of e-cigarette use. APACPH reports that some studies on the relative safety of e-cigarettes have been controversial due to conflicting interest and issues with research methods. In the Asian region, there is also a growing concern over the link between COVID-19 and e-cigarette use, with e-cigarette consumers at a greater risk of the infection and more severe morbidities.

(https://www.apacph.org/wp/2021/04/the-case-for-tightening-e-cigarette-regulations-in-southeast-asiahttps://www.apacph.org/wp/2021/04/the-case-for-tightening-e-cigarette-regulations-in-southeast-asia).[60]

### 5.3 Society of toxicology (SOT)

During the 2020 Society of Toxicology (SOT) annual meeting, a position paper on e-cigarette use was presented. The highlights are summarized below.

1. Cancer Risk Assessment: SOT has developed a scale to assess the potential cancer risk associated with e-cigarettes, ranging from a small fraction of the risk of traditional cigarettes to a risk exceeding half that of traditional cigarettes.
2. Long-Term Health Effects: Studies published by SOT highlight the lack of long-term data on the health effects of e-cigarettes and emphasize the potential for similar long-term cardiovascular and pulmonary risks as those associated with traditional cigarettes.
3. Exposure to Harmful Chemicals: SOT research has focused on the types of chemicals found in e-cigarette aerosols and their potential health consequences. Important Considerations:

a. E-cigarettes are not harmless: While they may be less harmful than traditional cigarettes, they are not a safe alternative.
b. Youth are particularly vulnerable: Nicotine addiction and potential brain development issues are serious concerns for young people who use e-cigarettes.
c. Long-term effects are still being studied: The full extent of the health impacts of e-cigarettes may not be known for years to come [63].

**Table 1:** Summary of some guidelines on E-cigarette use.

**International Regulatory Units**	**Guidelines**	**References**
European Commission Scientific Committee on Health, Environmental and Emerging Risks (EU-SCHEER)	Concerning the postulations that electronic cigarettes can serve as gateway to smoking/the initiation of smoking, particularly for adolescents, the SCHEER concludes that there is moderate evidence that e-cigarettes are a gateway to smoking for young people. However, there is strong evidence that nicotine component of e-liquids is linked to the development of addiction and that flavourings are pivotal in creating attractiveness to the use of e-cigarettes	[61]
The Asia Pacific Academic Consortium for Public Health (APACPH)	Judging from various countries in South-Eastern Asia, APACPH advocates caution in e-cigarette use and need for more research into the long-term cumulative effect of E-cigarette. The relationship between e-cigarette and COVID−19 incidence must be painstakingly looked into	https://www.apacph.org/wp/2021/04/the-case-for-tightening-e-cigarette-regulations-in-southeast-asia
Public Health England (PHE)	PHE concluded that the current best estimate is that e-cigarettes are around 95 % less harmful than smoking. Nearly half the population (44.8 %) don't realise e-cigarettes are much less harmful than smoking. There is no evidence so far that e-cigarettes are acting as a route into smoking for children or non-smokers	[62]; https://www.gov.uk/government/publications/e-cigarettes-an-evidence-update

### 5.4 Public health England (PHE)

The PHE has the following stipulations on E-cigarette use.

1. The current best estimate is that e-cigarettes are about 95 % less harmful than conventional smoking. Although, there has been an overall shift towards the inaccurate perception of e-cigarette being as harmful as cigarettes over the last year. This is in contrast to current expert estimates.
2. Smokers who have not succeeded using other smoking cessation schemes could be encouraged to try e-cigarettes to stop smoking. Also, encouraging smokers who cannot or do not want to stop smoking to switch to e-cigarette could help minimize reduce smoking-related pathologies and morbidities.
3. There is no scientific finding suggesting that e-cigarette use is compromising the long-term decline in conventional tobacco smoking among adults and youth. Recent findings support the Cochrane Review report that e-cigarette can help people to stop smoking and reduce their rate of smoking.
4. The appropriate and ethical use of e-cigarette does not present major risk of nicotine intoxication to users, but e-juice should be in ‘childproof' packaging. The precision of nicotine content information on the labels does not currently raise any major health concern. (https://www.gov.uk/government/publications/nicotine-vaping-in-england-2022-evidence-update.; https://www.gov.uk/government/publications/e-cigarettes-an-evidence-update.)

## 6 Who policy and guidelines On E-cigarette use

In 2023, WHO released a technical document on e-cigarette use titled: “*Electronic cigarettes: call to action”*. This preemptive approach on e-cigarettes suggests that country-specific policies should be documented and enacted. In addition to this, timely and strong decisive regulations to disallow the onset of e-cigarettes use particularly to protect children, as well as non-smokers and reduce negative health impact on the populace. Major highlights are enumerated below:

1. Countries with a ban on the sale of e-cigarettes, should reinforce actualization of the ban. This can be achieved via consistent and continuous monitoring to ensure compliance.
2. In countries where commercialization of e-cigarettes is allowed, there should be major regulations to reduce their harm to the population. This can be achieved by reducing their appeal via banning of flavourings, increase taxation and reduction in the concentration of nicotine.
3. Regardless of national ban or otherwise on permit of e-cigarette commercialization, major measures must be put in place to motivate and encourage current smokers of tobacco products to quit tobacco smoking through proven means including counselling from healthcare workers, mobile and digital cessation services and other approved therapeutic cessation interventions.
4. Countries should not encourage and embark on smoking cessation strategy that allows the sale of e-cigarettes as consumer products. Any tobacco cessation strategies using these products should carefully weigh national implications and the risk of uptake, and consider it as an option after other options have been exhausted. There must be a controlled clinical strategy to use e-cigarettes as a medical cessation plan rather than as a consumer product [64].

## 7 Recommendations and conclusion

About a decade ago, e-cigarettes were introduced into the market as a safer alternative to traditional tobacco smoking and a useful component of smoking cessation plan. However more research findings cutting across, in vitro, in vivo and some acute clinical investigations have not affirmatively provided research backing to this use. A key unanswered question, begging for scientific answer is whether e-cigarettes are a safer alternative to traditional smoking. The flavour component and nicotine content pose a huge neurological and behavioral concern especially in young adults. Putting these together, our perspectives are as follows:

1. There should be a national policy on the use of e-cigarette by health organization and health policy drivers.
2. There is need for more extensive study on the chronic effect of long-term exposure to e-cigarette use.
3. Major toxicological organizations have raised concerns especially with dual users i.e., those exposed to both traditional cigarette and e-cigarettes, due to the possible over-exposure and potentiating effects.
4. The health risks associated with e-cigarettes should be evaluated relative to those of cigarettes and also independently to identify any unique health risks posed by vaping device features and consumer behaviours.

Our review on the comparative effect of traditional tobacco smoking and e-cigarette consumption across major systems such as the respiratory, cardiovascular, gastrointestinal, reproductive, neurological and the immune system suggest that e-cigarette is not overall a safer alternative, acutely it poses more respiratory and cardiovascular toxicities, however, its chronic effect is largely unknown in both human and animal studies.

## CRediT authorship contribution statement

All authors contributed to the study concept and structure. The final draft was read and approved by the authors.

## Consent for publication

Not applicable

## Ethics approval and Consent to participate

Not applicable

## Declaration of Competing Interest

The authors declare that they have no known competing financial interests or personal relationships that could have appeared to influence the work reported in this paper.
